# Adolescents’ self-recognition of their facial profile: associations with wellbeing, self-esteem and sociodemographic factors

**DOI:** 10.1590/2177-6709.31.2.e2625306.oar

**Published:** 2026-06-22

**Authors:** Cristiane Braga MACHADO-SILVA, Saul Martins PAIVA, Jéssica Madeira BITTENCOURT, Leniana Santos NEVES, Cristiane Baccin BENDO

**Affiliations:** 1 Federal University of Minas Gerais, School of Dentistry, Department of Child and Adolescent Oral Health (Belo Horizonte/MG, Brazil).; 2 Federal University of Minas Gerais, School of Dentistry, Department of Restorative Dentistry (Belo Horizonte/MG, Brazil).

**Keywords:** Adolescent, Self concept, Personal satisfaction, Orthodontics, Dental esthetics, Adolescente, Autoimagem, Satisfação pessoal, Ortodontia, Estética dentária

## Abstract

**Objective:**

To examine the accuracy of adolescents’ self-recognition based on their facial profiles and determine the influence of sociodemographic characteristics and perceived well-being and self-esteem on this ability.

**Methods:**

A cross-sectional study was conducted with 158 adolescents (10-19 years old) who were either students or patients at the School of Dentistry, Federal University of Minas Gerais, Brazil. The facial profile of adolescent was photographed and included on a template of ten profile photographs, being nine standardized photographs and one was the participant’s own photograph. The adolescent was asked to recognize her/his own profile among these ten photographs. Perceived self-esteem was measured by the Brazilian version of the Rosenberg Self-Esteem Scale. A questionnaire addressed sociodemographic characteristics, with one item assessing whether the adolescent’s wellbeing was affected by her/his oral condition. Adjusted Poisson regression was performed (p<0.05).

**Results:**

Adolescents 17 to 19 years of age were 1.78 times more likely to recognize their own facial profile, compared to adolescents 10 to 13 years of age (95%CI:1.31-2.43). Adolescents’ perception that their general wellbeing was impacted by their oral conditions were less likely to recognize their own facial profile compared to those whose did not perceive this impact (PR = 0.72; 95% CI: 0.55-0.94).

**Conclusions:**

Older adolescents were more likely to recognize their own facial profile, while those reporting that oral health concerns affected their well-being showed poorer recognition ability. Engaging adolescents in decisions and considering their perceptions enhances satisfaction, adherence, and treatment outcomes.

## RESUMO

**Objetivo:**

Avaliar a precisão com que adolescentes reconhecem seus próprios perfis faciais e analisar como fatores sociodemográficos, bem-estar autopercebido e autoestima interferem nesse reconhecimento.

**Métodos:**

Foi realizado um estudo transversal com 158 adolescentes (10-19 anos), estudantes ou pacientes da Faculdade de Odontologia da Universidade Federal de Minas Gerais, Brasil. O perfil facial dos adolescentes foi fotografado e incluído em um modelo de 10 fotografias de perfil, sendo nove fotografias padronizadas e uma fotografia do próprio participante. Os participantes foram instruídos a identificar o seu próprio perfil facial dentre o conjunto de 10 fotografias. A autoestima percebida foi mensurada pela versão brasileira da Escala de Autoestima de Rosenberg. Um questionário abordou características sociodemográficas, com um item avaliando se o bem-estar do adolescente era afetado por sua condição bucal. Foi realizada regressão de Poisson ajustada (p<0,05).

**Resultados:**

Adolescentes de 17 a 19 anos apresentaram 1,78 vezes mais probabilidade de reconhecer seu próprio perfil facial, em comparação com adolescentes de 10 a 13 anos (IC 95%: 1,31-2,43). A percepção dos adolescentes de que seu bem-estar geral era afetado por suas condições bucais resultou em menor probabilidade de reconhecer seu próprio perfil facial, em comparação com aqueles que não percebiam esse impacto (RP=0,72; IC 95%: 0,55-0,94).

**Conclusões:**

Adolescentes mais velhos apresentaram maior probabilidade de reconhecer seu próprio perfil facial, enquanto aqueles que relataram preocupações com o bem-estar relacionado à saúde bucal demonstraram menor capacidade de reconhecer seu perfil. Engajar adolescentes nas decisões e considerar suas percepções melhora a satisfação, a adesão e os resultados do tratamento.

## INTRODUCTION

Adolescence is a phase of human development between 10 and 19 years of age involving growth and complex biopsychosocial development.^1^ This is an important period of dentofacial development, during which significant changes occur in face and dentition, such as the establishment of the permanent dentition and more rapid facial growth.^2^ Adolescents constitute a substantial proportion of orthodontic patients,^3^ driven by parental concerns regarding their children’s social acceptance and esthetic appearance,^4^ ^,^ ^5^ as well as the impact of dental aesthetics on adolescents’ self-esteem, social interactions, and interpersonal relationships.^6^ ^,^ ^7^

Individuals with poor dentofacial esthetics are susceptible to bullying in childhood, and see themselves as less attractive and less socially accepted.^7^ ^,^ ^8^ The search for orthodontic treatment among adolescents is motivated by the desire to improve their appearance and social acceptance.^6^

During orthodontic treatment, the facial profile can undergo substantial changes, depending on the treatment proposed. Thus, the adequate planning of treatment should include the patient’s participation based on a recognition of her or his profile. Assessing the extent of patients’ awareness of their facial profile is crucial to preventing misconceptions, managing unrealistic expectations, and enhancing communication between orthodontists and patients.^9^ Treatment planning should account for the adaptation and contour of soft tissues, which often impose limitations on orthodontic interventions and the patient’s esthetic expectations.^3^ ^,^ ^4^

The treatment plan should be a collaborative process that takes into account the patient’s needs, opinions, and expectations. It is essential that patients are properly informed about which treatment best meets their needs, with a clear understanding of both the problem and the purpose of the proposed treatment.^10^ This approach is aligned with the patient-centered care model and the principles of evidence-based dentistry. It is important to remember that the pursuit of orthodontic treatment is often related to aesthetic reasons, and this type of treatment can lead to significant changes in the facial profile. Therefore, it is crucial to understand how the patient self-recognizes his/her profile, in order to align expectations and outcomes. The literature indicates that older adolescents and females are more likely to self-recognize their facial profile.^3^ A study has shown that girls tend to be more critical and concerned with their dentofacial appearance compared to boys.^6^

Self-recognition is characterized by identifying their own profile from a set of different profiles, and differs from self-perception that is defined as selecting the profile most similar to their own among various profile types.^11^ However, most previous studies evaluated self-perception using constructed images and demonstrated that individuals have a limited perception of their own profile.^12^ ^-^ ^16^ Few studies have assessed self-recognition of facial profile,^3^ ^,^ ^11^ and none have been conducted in Brazil. Given the lack of scientific evidence on self-recognition of facial profile among Brazilian adolescents and the importance of this topic in orthodontic diagnosis and treatment planning, this study aimed to examine the associations of sociodemographic characteristics, overall well-being related to oral conditions, and self-esteem with self-recognition of facial profile among Brazilian adolescents.

## MATERIAL AND METHODS

This study was approved from the Human Research Ethics Committee of the Federal University of Minas Gerais (protocol number: 58928322.2.0000.5149). Written informed consent was obtained from the participants prior to the data collection process. Those less than 18 years of age signed a Term of Assent, the versions of which were drafted considering the age groups studied. 

A cross-sectional study was developed with adolescents aged 10 to 19 years old, from patients treated at the Federal University of Minas Gerais Clinics and students from the first period of the Undergraduate Course in Dentistry of the Federal University of Minas Gerais. A total of 158 adolescents (84 girls and 74 boys) between 10 and 19 years of age were included. This sample size was determined based on the feasibility and availability of participants during the study period. Adolescents with craniofacial anomalies or deformities and cognitive deficit or psychological disorders (based on the reports of parents/guardians) were excluded.

The power of the sample to compare two means was calculated with the OpenEpi program (The Open Projed, Atlanta, GA, USA), considering a 95% confidence interval and the difference in means of the self-perception of facial profile between age groups. The power of the sample was 93.2%. 

The facial profile of each participant was photographed using a Canon EOS Rebel T6i digital camera (Canon, USA) with an objective of 18-55 mm at a distance of 1 meter. Patients were photographed at the clinic where they were undergoing care. Undergraduate students were photographed in the classroom. A single orthodontist took all images. The photographs were taken with the participant seated and the head in its natural position.^17^ The image was subsequently placed completely in black and white, using the PowerPoint software (Microsoft, Remond, WA, USA), as described in a previous study.^18^

**Figure 1:**
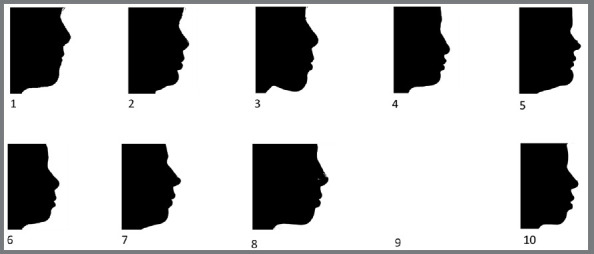
Example of facial profile template presented to the participants.

The templates were composed of various silhouette profiles of different patients to standardize the recognition process. The profiles were selected from orthodontic records at a private office after authorization from patients. Profiles of different ethnicities were selected and were darkened with the same process performed on the photographs of the participants, resulting in silhouette images. Six templates with nine photographs were created considering differences in sex and age: 10-to-13-year-old girls; 10-to-13-year-old boys; 14-to-16-year-old girls; 14-to-16-year-old boys; 17-to-19-year-old girls; and 17-to-19-year-old boys. The templates were created following the model described by Varatharaju et al.^3^ The arrangement of the photographs in the templates was standardized, with two rows: the upper row containing five profile silhouettes and the lower row containing four silhouettes, with position #9 left empty. Position #9 was completed with the participant’s profile photograph (Fig. 1).

The dependent variable was the self-recognition of facial profile. It was investigated through the presentation of a template. After inserting the photograph of the participant’s facial profile into the corresponding template (according to age group and gender), the adolescent was asked to identify their facial profile among the ten presented in the template. This variable was dichotomized as 0 (unable to recognize one’s own profile) and 1 (able to recognize one’s own profile).

The independent variables included individual and socioeconomic characteristics of the adolescents, as well as their self-esteem and whether their overall well-being was affected by their teeth. These variables were collected through questionnaires administered to both parents/guardians and adolescents.

Self-esteem was measured using the validated Brazilian version of the Rosenberg Self-Esteem Scale^19^, translated from Rosenberg’s original scale^20^, using the overall score. This scale is composed of 10 positive or negative statements related to feelings of self-esteem and self-worth. Each item is scored on a four-point Likert scale with the following response options: strongly agree = 4; agree = 3; disagree = 2; strongly disagree = 1. For the negative statements (items 3, 5, 8, 9, and 10), the scores were reversed. Subsequently, the total score was obtained by summing all 10 items. The overall score was dichotomized as ‘Low self-esteem’ or ‘High self-esteem”. 

A pilot study was carried out as part of the training to operationalize data collection, and was conducted with 16 adolescents. The templates and questionnaires developed were tested. As the results of the pilot study indicated no need for methodological adjustments, the participants were included in the main study.

## STATISTICAL ANALYSIS

Statistical analysis was performed using the IBM SPSS Statistics (SPSS Statistics for Windows, Version 25.0. Armonk, NY, USA). The data were submitted to descriptive statistics (frequencies, mean, and standard deviation). Unadjusted Poisson regression analyses with robust variance were performed between self-recognition of facial profile and independent variables. Independent variables were introduced into the adjusted model based on their statistical significance (p<0.20) and/or clinical epidemiological importance. Adjusted Poisson regression analyses with robust variance were conducted, with the significance level set at 5% (p<0.05). Missing data in self-reported variables were handled using a complete-case approach in the regression analysis.. Participants with missing information in any of the variables included in the analyses were excluded from the analytical sample.

## RESULTS

The sample was composed of 158 adolescents. Most of them (n=84; 53.3%) were girls, 93 (59.6%) were self-declared as non-white, 83 (53.9%) did not have their wellbeing affected by their oral conditions according to the reports of the parents/guardians, and 108 (75.5%) had a family income greater than the monthly minimum wage. Mean age of the adolescents was 14.64 (± 2.614) years. Table 1 displays the sociodemographic characteristics of the participants.

**Table 1:** Individuals and socioeconomic characteristics of sample (n=158).

Variables	Frequency (%)
Sex	
Female	84 (53.3)
Male	74 (46.8)
Age	
17 to 19 years	44 (27.8)
14 to 16 years	57 (36.1)
10 to 13 years	57 (36.1)
Self-declared skin color *	
White	63 (40.4)
Non-White	93 (59.6)
Family income *	
≤ monthly minimum wage	35 (24.5)
> monthly minimum wage	108 (75.5)
Rosenberg Self-Esteem Scale *	
Low self-esteem	68 (46.6)
High self-esteem	78 (53.4)
Wellbeing affected *	
No	83 (53.9)
Yes	71 (46.1)

* Samples with missing data for self-reported skin color, family income, Rosenberg Self-Esteem Scale and wellbeing.

The unadjusted analyses demonstrated associations between the recognition of one’s own facial profile and both age 17 to 19 years (PR=1.74; 95%CI: 1.28- 2.37; p<0.001) and wellbeing not affected by oral conditions (PR=0.74; 95%CI: 0.57-0.97; p=0.031). Self-esteem was not associated with recognition of one’s own facial profile (PR=0.89; 95%CI: 0.68-1.16, p=0.368). However, because of its clinical epidemiological importance in the model, this variable was included in the adjusted analysis (Table 2). 

**Table 2:** Results of unadjusted analysis of associations between recognition of one’s own facial profile and individual and socioeconomic characteristics

Variable	Recognized profile		Unadjusted PR (95% CI)	p-value
	No n (%)	Yes n (%)		
Sex				
Female	20 (28.2)	51 (71.8)	1.27 (0.98-1.66)	0.072
Male	27 (43.5)	35 (56.5)	1.00	
Age				
17 to 19 years	5 (12.8)	34 (87.2)	1.74 (1.28- 2.37)	<0.001
14 to 16 years	18 (39.1)	28 (60.9)	1.22 (0.85- 1.76)	0.292
10 to 13 years	24 (50.0)	24 (50.0)	1.00	
Family income				
≤ monthly minimum wage	14 (43.8)	18 (56.3)	0.81 (0.58-1.13)	0.211
> monthly minimum wage	27 (30.3)	62 (69.7)	1.00	
Self-declared skin color				
White	19 (34.5)	36 (65.5)	1.04 (0.80-1.34)	0.786
Non-White	28 (36.8)	48 (63.2)	1.00	
Rosenberg Self-Esteem Scale				
Low self-esteem	23 (39.7)	35 (60.3)	0.89 (0.68-1.16)	0.368
High self-esteem	21 (31.8)	45 (68.2)	1.00	
Wellbeing affected				
Yes	27 (45.0)	33 (55.0)	0.74 (0.57-0.97)	0.031
No	18 (26.1)	51 (73.9)	1.00	

Poisson regression analyses; PR = prevalence ratio; CI = confidence interval; p = probability; Statistically significant association = p<0.05.

Table 3 displays the results of the adjusted analysis. Adolescents 17 to 19 years of age were 1.78 times more likely to recognize their own facial profile compared to adolescents 10 to 13 years of age (PR=1.78; 95%CI: 1.31-2.43; p<0.001). Participants whose general wellbeing was affected by their oral conditions were less likely to recognize their own profile, compared to those whose wellbeing was not affected (PR=0.72; 95%CI: 0.55-0.94; p=0.016). 

**Table 3:** Results of adjusted analysis of association between recognition of one’s own facial profile and independent variables.

Variables	Adjusted PR (95% CI)	p-value
Sex		
Female	1.19 (0.93-1.53)	0.174
Male	1.00	
Age		
17 to 19 years	1.78 (1.31-2.43)	<0.001
14 to 16 years	1.26 (0.87-1.84)	0.225
10 to 13 years	1.00	
Wellbeing affected		
Yes	0.72 (0.55-0.94)	0.016
No	1.00	
Rosenberg Self-Esteem Scale		
Low self-esteem	0.95 (0.73-1.23)	0.687
High self-esteem	1.00	

Poisson regression analyses; PR = prevalence ratio; CI = confidence interval; p = probability; Statistically significant association = p<0.05.

## DISCUSSION

This study contributes to the existing literature on orthodontic diagnosis and treatment planning, by providing new insights into Brazilian adolescents’ self-recognition of facial profiles and its association with sociodemographic characteristics and well-being. This study is innovative in its use of templates derived from various real facial profiles representative of the Brazilian population. Additionally, the creation of six templates spanning different age ranges and sexes allowed for comprehensive coverage of the entire adolescent period. Furthermore, using a visual representation of the participants own facial profile through a photograph allowed for the evaluation of self-recognition rather than self-perception. Self-recognition involves identifying one’s own body parts and appearance, while self-perception includes how individuals interpret their behaviors, emotions, integrating both physical and psychological aspects.^21^ ^,^ ^22^

In the present study, older adolescents whose wellbeing was not affected by their oral conditions were more likely to recognize their own facial profile. With regards to age, the results are consistent with findings from other studies, which indicate that older adolescents are more likely to recognize their own facial profile than younger adolescents.^3^ ^,^ ^23^ A previous study suggest that age range 15 to 22 years encompasses a period during which social acceptance and appearance are particularly significant.^23^ Adolescents in this stage of development are increasingly aware of flaws and imperfections in their peers and exhibit a heightened ability to recognize and relate to similar aspects within themselves.^24^ These issues should be taken into account when planning the orthodontic treatment for these patients. Understanding how age is associated with the recognition of one’s own facial profile contributes to higher patient satisfaction when planning is performed with the active participation of the patient.^9^

No significant association was found between sex and recognition of one’s own facial profile in the present study, although girls tended to recognize their facial profile more than boys. A previous study demonstrated that girls have greater self-recognition and hypothesized that this may be because sexual maturity occurs earlier in girls.^3^ Moreover, girls are more critical and worried about their dentofacial appearance than boys.^6^

Good oral health status and higher levels of education are determinants of a positive perception of orofacial esthetics and patients with these characteristics tend to be more concerned with their orofacial appearance.^25^ This leads to a better understanding of the present results, which demonstrated that adolescents whose wellbeing was not affected by their oral conditions were more likely to recognize their own profile compared to those whose wellbeing was affected. 

No significant association was found between self-recognition of one’s facial profile and the self-esteem in this study. A previous study involving adolescents and young adults, also utilizing the Rosenberg Self-Esteem Scale, reported similar findings, showing no association between facial profile and self-esteem. This suggests that individuals may have limited awareness of their head shape.^26^ Furthermore, another previous study with adolescents demonstrated that the facial profile did not impact oral health-related quality of life^27^, and this finding may reflect the reduced aesthetic impact of the facial profile in a frontal view, which is the perspective through which individuals primarily perceive themselves in daily life.^27^ Self-esteem is a multifaceted construct, and literature indicates that it is more strongly influenced by psychological factors than by craniodentofacial characteristics.^26^ ^,^ ^27^

This study has some limitations. The sample was recruited from a single site, which may limit the generalizability of the findings. Additionally, the lack of information on participants’ previous orthodontic treatment may have influenced the awareness of their facial profile. Furthermore, the assessment of the impact of oral health conditions on well-being was conducted using a single-item measure, which may not fully capture the multidimensional nature of this construct, limiting the sensitivity of the findings. Furthermore, for participants under 18 years of age, information was obtained through parental/guardian reports, which may introduce discrepancies between the proxy’s perception and the child’s or adolescent’s actual experience. Therefore, results related to well-being should be interpreted with caution. Future studies are encouraged to use validated multidimensional instruments and, whenever possible, prioritize direct self-reporting by participants. However, it was based on self-reports among those 18 years of age and older. Future studies should use instruments in which only adolescents report this information. Furthermore, although the sample included both patients and dentistry students, it should be noted that the participating students were in their first semester of dental school. Therefore, their familiarity with facial esthetic analysis is likely more comparable to that of the general population than to that of senior clinicians. Nonetheless, differences in baseline knowledge and awareness of oral and facial conditions between these groups may still have influenced self-recognition and perception outcomes. Consequently, the findings should be interpreted with caution, and future studies are encouraged to consider stratified analyzes or more homogeneous samples. This study has some strengths, as the use of a validated instrument considered the ‘gold standard’ for measuring self-esteem (Rosenberg Self-Esteem Scale).^28^ Another strength was the evaluation of self-recognition rather than self-perception, based on templates created from photographs of real individuals. This represents an advance over most of previous studies, which assessed self-perception using profile images generated with computer graphics.^12^ ^,^ ^16^ The present study used profiles from various real patients to create the templates, ensuring that ethnic differences and age would not influence the adolescents’ recognition of their facial profiles. The present study also included participants between 10 to 19 years of age, covering the entire period of adolescence.^1^

The search for orthodontic treatment mainly occurs due to esthetic issues and such treatment can lead to significant changes in the facial profile.^3^ Moreover, perceptions of facial esthetics differ between laypersons and orthodontists.^16^ ^,^ ^25^ ^,^ ^29^ Thus, the present findings underscore the importance of the adequate planning of orthodontic treatment with the participation of patients, who have opinions and concerns with regards to their own appearance that orthodontists should take into account. This practice is in line with evidence-based dentistry and patient-centered care, and can improve the dentist-patient relationship, increase patient adherence to treatment, and assist in the development of healthy habits, such as adequate oral hygiene and nutrition. ^10^ ^,^ ^30^ The present study shows the relevance of the subjective characteristics and age of each individual in health outcomes. Taking such factors into account contributes to patient satisfaction and wellbeing.

## CONCLUSIONS

Older adolescents were more likely to recognize their own facial profile, while those reporting that oral health concerns affected their well-being showed poorer recognition ability. 

By identifying which aesthetic and functional concerns are most relevant to each patient, the clinician can tailor their communication, establish more realistic and individualized treatment expectations, and align explanations with the patient’s own priorities. This approach can also promote adherence to appliance use and oral hygiene care. Furthermore, incorporating patient perceptions into the clinical assessment can support the personalization of treatment planning, including prioritizing treatment goals and continuously monitoring concerns reported throughout follow-up.

## Data Availability

Due to the sensitive nature of the data and ethical restrictions, the datasets supporting this study are not publicly available.
